# Egyptian practical guidance in lipid management 2020

**DOI:** 10.1186/s43044-021-00140-1

**Published:** 2021-02-23

**Authors:** Hesham Salah El Din Taha, Hala Mahfouz Badran, Hossam Kandil, Nabil Farag, Abbas Oraby, Magdy El Sharkawy, Khaled Shokry, Fouad Fawzy, Hossam Mahrous, Juliette Bahgat, Mina Samy, Mirna Mamdouh Shaker

**Affiliations:** 1 Department of Cardiology, Faculty of Medicine, Cairo University, 27 Nafezet Sheem El Shafae St Kasr Al Ainy, Cairo, 11562 Egypt; 2 Menoufia University, Shebeen El-Kom, Egypt; 3 Ain-Shams University, Cairo, Egypt; 4 Suez Canal University, Ismailia, Egypt; 5 Military Forces, Cairo, Egypt

**Keywords:** Dyslipidemia, Practical guidance, Atherosclerotic cardiovascular disease

## Abstract

**Background:**

Numerous epidemiological investigations and randomized clinical studies have determined that dyslipidemia is a major contributor to atherosclerotic cardiovascular disease (ASCVD). Consequently, the management of serum cholesterol and low-density lipoprotein levels has become a central objective in the effort to prevent cardiovascular events.

**Main body:**

Many guidelines were issued by different organizations and societies to define patient risk and establish important recommendations for management strategies. Newer cholesterol-lowering agents (non-statin drugs) are described, and their use is directed primarily to secondary prevention in patients at very high risk of new ASCVD.

**Conclusion:**

The present guidance summarizes the current methods for risk estimation and outlines the most recent data on lipid management in a simple user-friendly format, to improve physician awareness and help implement guidelines in the daily practice.

## Background

Recently, World Health Organization (WHO) reported that cardiovascular diseases (CVD) account for 46% of total deaths in Egypt. Atherosclerotic cardiovascular diseases (ASCVD) are a key public health problem with considerable social and economic consequences in terms of healthcare demands, lost efficiency, and premature mortality [1]. Egypt is having a high incidence of early atherosclerotic cardiovascular events. The incidence of CVD is fast-shifting to the youth, a trend that is specifically prevalent in the capital city of Cairo and the underserved urban societies, where adoption of unhealthy lifestyles, fast food-eating habits, and sedentary lifestyles are an increasing reality [1–3]. Dyslipidemia is a major risk factor for CVD, and researches have demonstrated that 37% of the Egyptian population has elevated blood cholesterol levels with an overall target accomplishment of only 34.4% [4, 5]. Multiple risk factors are usually involved in the development of ASCVD; therefore, assessment of total cardiovascular (CV) risk becomes imperative.

## Main text

**Fig. 1:**
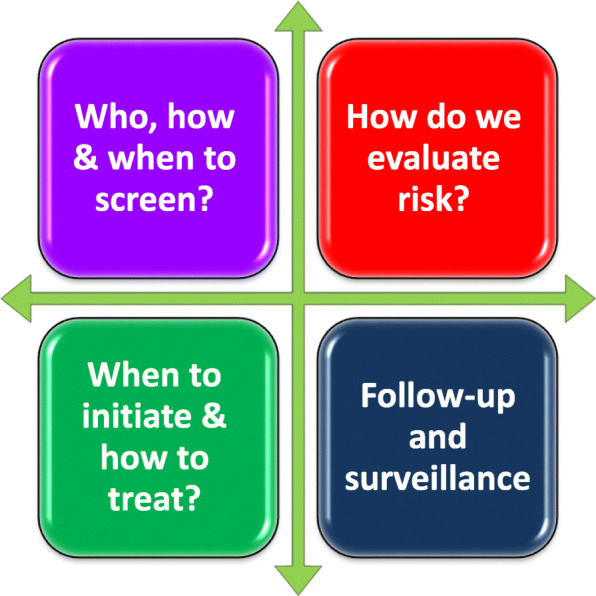
Approach to lipid management

The rationale of this consensus is to provide the most recent data on lipid management in a simple user-friendly format, improve physician awareness, and help implement guidelines. It emphasizes the practical aspects in lipid management, answers commonly asked valuable questions on dyslipidemia, and contributes to reducing CV onus in Egypt (Fig. 1).

### Who and when should we screen?

#### Visiting a physician is always an opportunity for checkup

Screening for CV risk including complete lipid profile is recommended for all adults ≥ 40 years, at any age in individuals having ASCVD, e.g., coronary artery disease (CAD), cerebrovascular disease, peripheral arterial disease (PAD), or risk factors like diabetes mellitus (DM), hypertension, obesity, and smoking. Screening should be considered as early as age 2 years in persons with a family history of early CVD or familial hypercholesterolemia (FH); it can be considered in adults at the age of 20 years or more and not on lipid-lowering therapy [1–6].

### How should we screen?

Lipid testing can generally be done non-fasting, while fasting lipid profile is recommended for individuals with triglycerides (TG) > 400 mg/dL, or having a history of familial dyslipidemia. In addition to lipid profile testing, the risk evaluation must include history taking and physical examination including blood pressure measurement. Lipid profile should include total cholesterol (TC), low-density lipoprotein cholesterol (LDL-C), high-density lipoprotein cholesterol (HDL-C), TG, and non-HDL-C. Apolipoprotein B (Apo B) is advised for the risk evaluation especially in individuals with high TG, DM, obesity or metabolic syndrome, or very low LDL-C. Lipoprotein (a) [Lp(a)] should be taken into account at least once in life, and in certain patients with positive family history of premature CVD [1–3].

### How to assess CVD risk?

According to the latest guidelines, risk assessment is based on country-specific risk charts. The most available and commonly used one is the European SCORE (Systematic Coronary Risk Estimation) system. It is reconfigured for use by adapting for materialistic changes in CVD deaths and risk factor occurrence [1, 4–6]. In Egypt, it is recommended to use the high-risk SCORE chart, knowing that this may underestimate the risk in very-high-risk countries like ours, having a cardiovascular disease mortality rate > 350/100,000 [1].

Total CV risk estimation is advised for asymptomatic adults > 40 years without evidence of CVD, DM, chronic kidney disease (CKD), FH, and LDL-C > 190 mg/dL, as these categories are at high or very high risk and are not requiring risk scoring. Arterial (carotid and/or femoral) plaque load on the ultrasound and coronary calcium score assessment on computed tomography (CT) might be considered as risk changers in persons at low or moderate risk [7]. Therapeutic objectives are based on the risk point (the higher the risk, the lower the LDL-C goal), as shown in (Table 1) [1].

**Table 1:** Risk categories and LDL-C goals

Cardiovascular risk categories		LDL-C goal
**Very high risk**	1. Atherosclerotic cardiovascular disease (ASCVD) (clinical or imaging) 2. SCORE equal to or more than 10% 3. Family history of ASCVD or any major risk factor 4. Severe chronic kidney disease (estimated glomerular filtration rate < 30 mL/min) 5. Diabetes mellitus and or target organ damage: ≥ 3 major risk factors; or early onset of type 1 diabetes mellitus and long duration (> 20 years)	**< 55 mg/dL**
**High risk**	1. SCORE ≥ 5% and < 10% 2. Markedly elevated single risk factors, in particular TC > 310 mg/dL or LDL-C > 190 mg/dL or BP ≥ 180/110 mmHg 3. FH without other major risk factors 4. Moderate CKD (eGFR 30–59 mL/min) 5. DM without target organ damage, with DM duration ≥10 years or other additional risk factors	**< 70 mg/dL**
**Moderate risk**	1. SCORE equal or more than 1% and less than 5% 2. Young diabetic patients (type 1 < 35 years, type 2 < 50 years) with diabetes duration < 10 years in absence of other risk factors	**< 100 mg/dL**
**Low risk**	1. SCORE < 1%	**< 116 mg/dL**

### When to initiate and how to treat?

Management is always based on the level of risk. Lifestyle advice is recommended for all individuals regardless of their cardiovascular risk. In patients who are at low or moderate risk with LDL-C levels above the respective goals (but < 190 mg/dL), a 3-month trial of lifestyle intervention is recommended followed by re-assessment of lipid profile [8–10].

### What are the LDL-C goals?

In both primary and secondary preventive measures for individuals at extremely high risk, a therapeutic program that achieves ≥ 50% LDL-C reduction from baseline and an LDL-C goal of < 55 mg/dL are recommended. In patients at high risk, a treatment regimen that achieves ≥50% LDL-C reduction from baseline and an LDL-C goal of < 70 mg/dL is recommended. In individuals at moderate risk, an LDL-C goal of < 100 mg/dL should be taken into consideration. In persons who are at low risk, an LDL-C goal < 116 mg/dL may be considered [1].

### What are the non-HDL goals?

Non-HDL-C secondary goals are < 85, 100, and 130 mg/dL for very high-, high-, and moderate-risk people, respectively [1].

### What is the therapeutic regimen?

It is highly advised that a maximum tolerated dose of statin is uptitrated to achieve the goal level specific for each category of risk. If the maximum allowed dose of statin does not achieve the required levels, a combination with ezetimibe is highly recommended. In patients at very high risk not achieving their goal on a maximum tolerated dose of a statin and ezetimibe, adding a proprotein convertase subtilisin/kexin type 9 (PCSK9) inhibitor is encouraged. If a statin-based program will not be tolerated at any dosage (even after re-challenge), ezetimibe should be taken into consideration. PCSK9 inhibitor, added to ezetimibe, may also be considered to reach the LDL target level [1, 10, 11]. We endorse the recommendations of the Egyptian Association of Vascular Biology and Atherosclerosis (EAVA) on the usage of PCSK9 inhibitors in addition to statins ± ezetimibe in patients with ASCVD, by definition at very high risk; patients with ASCVD at very high risk who do not tolerate appropriate doses of at least three statins; and familial hypercholesterolemia patients with clinically diagnosed ASCVD, at very high CV risk [12].

### Management of dyslipidemia in different clinical settings

#### Elderly

For primary prevention, statin therapy is recommended for older individuals, > 75 years, according to the risk level (as mentioned in Table 1). Introduction of statin therapy for primary prevention in this fragile group may be considered if at a high risk or above. It is recommended that the statin is started at a low dose especially if there is substantial renal impairment and/or there is potential for drug interactions, and then up titrated upwards to accomplish the LDL-C treatment goals [1–3].

#### Children and adolescents

In children and adolescents, it is advised to deepen lifestyle therapy in those who suffer from lipid disorders related to obesity, involving moderate caloric restriction and regular aerobic physical activity [13]. However, it is reasonable to start statin in children and adolescents ≥ 10 years of age if LDL-C level is persistently ≥ 190 mg/dL or ≥ 160 mg/dL with a clinical presentation consistent with FH, and if they do not respond adequately with 3 to 6 months of lifestyle therapy [6].

#### Women

**Fig. 2:**
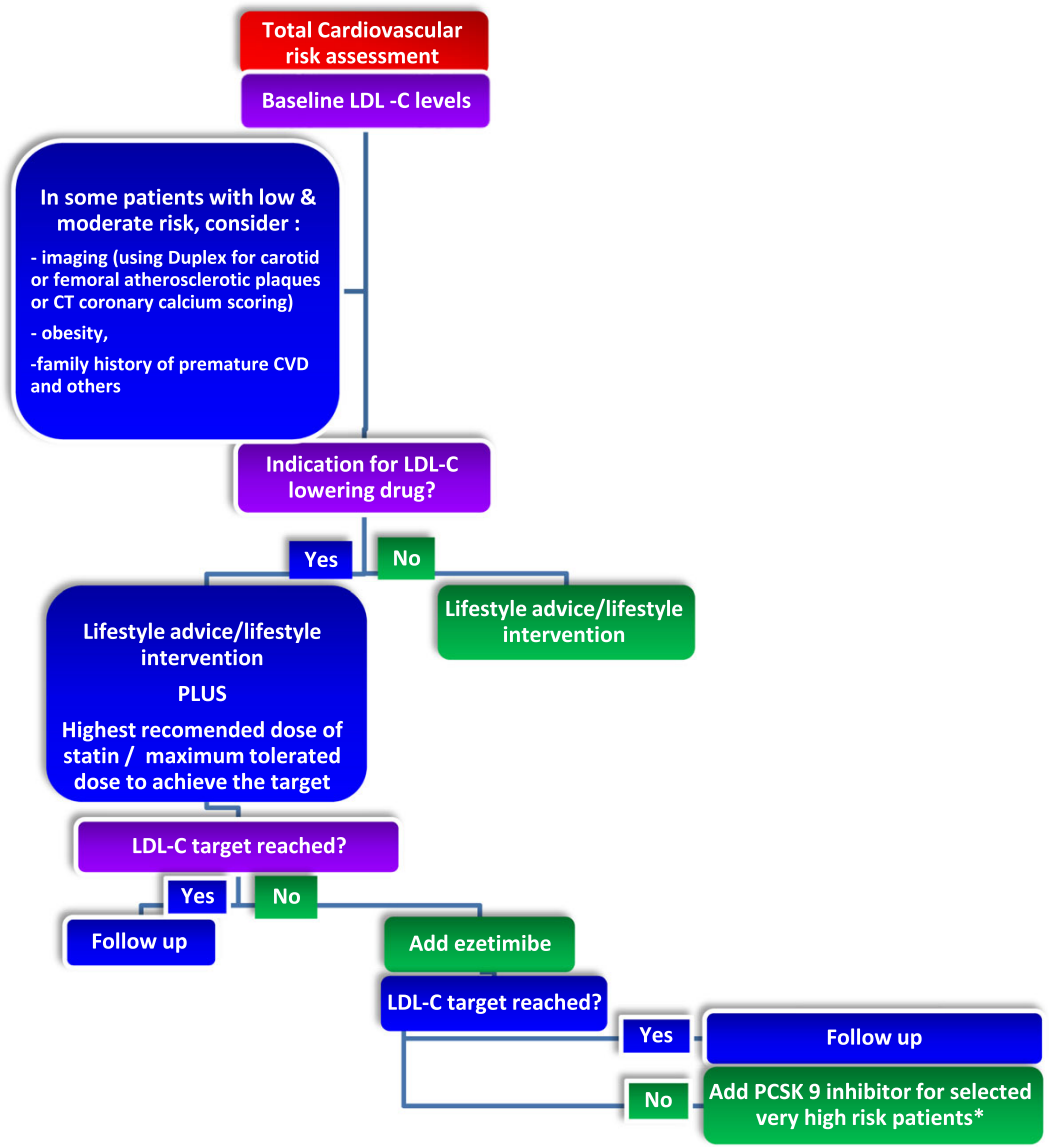
Treatment algorithm for LDL-C lowering. *Add PCSK9 inhibitor for secondary prevention in very-high-risk patients and for primary prevention in patients with FH and another major risk factor (very high risk). *Consider adding PCSK9 inhibitor for primary prevention patients at very high risk, but without FH

Lipid-lowering therapy should not be given 1 to 2 months before pregnancy is attempted, during pregnancy or breast-feeding. However, for severe FH, refer to a lipid specialist. Women of reproductive age who are being treated with statin and are sexually involved should be encouraged to use a credible form of contraception. Lipid-lowering therapy is highly recommended for primary and secondary prevention in women with the same target levels as men [1] (Fig. 2).

#### Acute coronary syndromes

Reda et al. [14] in phase II of the Egyptian cross-sectional CardioRisk project showed that premature ACS is prevalent in Egypt with 43% of men aged less than 55 years, and 67% of women under 65 years suffering from the disease, hence, the importance of treating the underlying risk factors.

It is recommended to initiate high-intensity statin therapy as soon as possible, irrespective of baseline LDL-C values, in all ACS patients not having contraindications or definite intolerance history. Lipid levels should be re-checked 4–6 weeks following ACS to establish whether the LDL-C target < 55 mg/dL has been achieved. If the LDL-C goal is not achieved after 4–6 weeks despite maximally tolerated statin therapy, adding ezetimibe is recommended. In patients in whom LDL-C goal is not achieved in spite of maximally tolerated statin therapy and ezetimibe, as well as in patients with statin contraindication or intolerance, adding PCSK9-inhibitor is recommended.

In patients undergoing percutaneous coronary intervention (PCI), regular treatment with high-dose statin (or loading on a background of chronic therapy) should be taken into consideration for primary or elective PCI. For patients with ASCVD who experience a second vascular event within 2 years (not necessarily of the same type as the first event) while taking maximally tolerated statin-based therapy, an LDL-C goal of < 40 mg/dL may be considered [15–17].

#### Stroke

In the general population, high TC is associated with higher risk of ischemic stroke. A meta-analysis of 26 trials that included > 90,000 patients found that statins reduced the risk of all strokes by approximately 21% [18]. Patients with a history of ischemic stroke or transient ischemic attack (TIA) are at very high risk of recurrent ASCVD, and it is recommended that they receive intensive LDL-C-lowering therapy to reduce the risk of recurrent stroke, myocardial infarction (MI), and vascular death [1].

#### Peripheral arterial disease

Patients with PAD are at very high risk of ASCVD. They are strongly advised to receive high-intensity LDL-C lowering therapy to reduce the risk of CV events, worsening of claudication, and to improve walking performance [1].

#### Diabetes mellitus

Diabetic dyslipidemia involves a triad of elevation in the level of TG, with decrease in HDL-C, and formation of small dense LDL particles [19]. Diabetic patients cannot be at low risk; they are at either very high, high, or at least moderate risk, as shown in (Table 2). The primary target in patients with DM remains LDL-C [1, 20]. However, due to the malignant nature of diabetic dyslipidemia that is not always revealed by elevation of LDL-C, secondary targets like non-HDL-C are particularly important in the diabetic population [21].

**Table 2:** Risk stratification for DM patients

**Very high risk**	Patients with DM and established CVD ***or*** with target organ damage ***or*** with 3 or more risk factors ***or*** T1DM with early onset (duration > 20 years)
**High risk**	Patients with DM without organ damage, with duration ≥ 10 years or any other additional risk factor
**Moderate risk**	Young patients (T1DM < 35 years, T2DM < 50 years), with DM duration ˂ 10 years without other risk factors

In patients with T2DM at very high risk, an LDL-C reduction of ≥ 50% from baseline and an LDL-C goal of < 55 mg/dL are recommended. In patients with T2DM at high risk, an LDL-C reduction of ≥ 50% from baseline and an LDL- C goal of < 70 mg/dL are recommended. Statins are recommended in patients with T1DM who are at high or very high risk. Statins are the main stay of therapy in diabetic patients; however, if the target LDL-C is not reached, combination therapy with ezetimibe is recommended. It is reasonable to give a combination of statin and ezetimibe from the start in very-high-risk patients. In patients at very high risk, with persistent high LDL-C despite treatment with a maximally tolerated statin dose, in combination with ezetimibe, as well as in patients having statin intolerance, a PCSK9 inhibitor is recommended. Lifestyle intervention is recommended (with a focus on weight reduction, and decreased consumption of fast-absorbed carbohydrates and alcohol). Icosapent should be considered in patients with high TG levels (above 200 mg/dL). Fibrates may be added in patients with low HDL-C and high TG levels (above 200 mg/dL) who are at high risk [1–4].

#### Chronic kidney disease

In the adult population, decreasing GFR is associated with increased CVD risk, independent of other risk factors. In the early stages of CKD, TG and Lp (a) readings are high, while HDL-C levels are low. LDL is changed into small dense LDL particles. Patients with stages 3a and 3b CKD (eGFR 30–59 mL/min/1.73m^2^) are considered to be at high risk of ASCVD, while those with stages 4 and 5 CKD (eGFR < 30 mL/min/1.73m^2^) are considered to be at great risk of ASCVD. The usage of statins or statin/ezetimibe combination is recommended in non-dialysis-dependent stages 3–5 CKD. In patients who are on statins, ezetimibe, or a statin/ezetimibe combination before dialysis commencement, the continuation of these drugs must be taken into account, particularly in patients with evidence of ASCVD. In dialysis-dependent CKD patients, who have ASCVD, the introduction of lipid-lowering medications should be considered. Expert opinion of a cardiologist and a nephrologist is advised for post-renal transplant and dialysis-dependent CKD patients [1–6] (Table 3).

**Table 3:** Statin doses in CKD patients

Statin	Maximum dose in CKD patients
Atorvastatin	**80 mg**
Rosuvastatin	**10 mg**
Simvastatin	**40 mg**
Pravastatin	**20 mg**

#### Familial dyslipidemias

Heterozygous familial hypercholesterolemia (HeFH) is a relatively common disorder. Its prevalence in the general population is estimated to be 1/200–250. Homozygous FH (HoFH) is a rare and life-threatening disease with a frequency of 1/160,000–1/320,000. FH causes premature CVD, with at least10-fold increased risk, due to lifetime very high plasma levels of LDL-C. The diagnosis of FH is usually based on clinical presentation. The Dutch Lipid Clinic Network (DLCN) is the commonly used criteria [15, 16, 22]. The diagnosis can be confirmed by showing relevant mutations in the pathogenic genes. FH should be *suspected* if LDL-C is > 190 mg/dL, there is history of premature CAD in the patient or a family member, there are tendon xanthomas in the patient or a family member, or there is history of sudden premature cardiac death of a family member [1, 23, 24].

*Other familial dyslipidemias* include familial combined hyperlipidemia (FCH) which is a very common mixed dyslipidemia (prevalence is 1:100–200) characterized by elevated concentrations of LDL-C, TG, or both and is an important cause of premature CAD. Familial dysbetalipoproteinemia is a rare inherited disorder and occurs due to genetic disorder for the E2 isoform of Apo E [1].

It is strongly recommended that FH need to be detected using clinical criteria, and verified via DNA analysis. Once the diagnosis of the index patient is established, family screening becomes essential. Patients with FH and ASCVD or who have another major risk factor are treated as being at very high risk, to achieve ≥ 50% reduction from baseline and an LDL-C < 55 mg/dL, while patients with no prior ASCVD or other risk factors are treated as being at high-risk. If LDL-C target is not achieved, a drug combination is recommended. Similar approach and target are recommended in FH patients at very high-risk group if primary prevention is proposed. Treatment with a PCSK9 inhibitor is recommended in very high-risk FH patients if the treatment goal is not attained on maximally tolerated statin plus ezetimibe [1].

### Management of hypertriglyceridemia

Hypertriglyceridemia (HTG) prevalence in adults is 10%.(25) HTG may be primary due to genetic causes or secondary to many causes such as increased alcohol consumption, obesity, metabolic syndrome, DM, hypothyroidism, renal disease, pregnancy, systemic lupus erythematosis (SLE), and medications (including corticosteroids, oral estrogen, tamoxifen, thiazides, non-cardio-selective beta-blockers, and bile acid sequestrants).(26) Fasting samples remain the method of choice. For non-fasting samples, TG > 175 mg/dL should be flagged as abnormal, while for fasting samples, abnormal concentrations correspond to TG > 150 mg/dL. Severe HTG, defined as plasma TG > 500 mg/dL is less common. Very severe HTG is defined as TG > 1000 mg/dL. The effect of LDL-C-lowering drugs such as statins, ezetimibe, and PCSK9 inhibitors on TG levels is usually modest (5–15%). Fibrates, omega-3-fatty acids, and niacin have more profound effects (25–45%). Although LDL-C-lowering drugs have only moderate effects on TG levels, they reduce ASCVD risk in patients with and without HTG. The most important principle for treating individuals with HTG is to manage lifestyle factors associated with HTG including increased alcohol consumption, DM, and obesity [1, 25, 26].

#### Recommendations

It is recommended to exclude and treat secondary causes of HTG [TG levels > 200 mg/dL]. Patients with HTG and at high risk must receive statin as a first-line treatment to reduce CVD risk. In high-risk (or above) patients with TG levels between 135 and 499 mg/dL despite statin treatment, n-3 PUFAs (icosapent ethyl 2 × 2 g/day) should be considered in combination with a statin. In high-risk patients who are at LDL-C goal with TG levels > 200 mg/dL, fenofibrate or bezafibrate might be considered in combination with statins. In selected primary prevention patients who are at low to moderate CV risk with TG levels > 200 mg/dL, omega-3 fatty acids may be considered [1].

#### Lifestyle goals for cardiovascular disease prevention

Carbohydrate intake should comprise 45 to 55% of the total energy intake. Intake of fruits, vegetables, legumes, nuts, whole grain cereal foods, dietary fibers, and fish is encouraged. Foods rich in trans-fats, alcohol consumption and exposure to smoking in any form should be avoided. Foods rich in saturated fats should not exceed 10%. Mono-unsaturated fat and poly-unsaturated fat should be used instead of saturated fats. The salt intake should be reduced to < 5 g/day. Moderately vigorous physical activity 30 to 60 min in most days is advised. Reduction of body weight should be advised (preferred body mass index (BMI) of 20 to 25 kg/m2, and waist circumference of < 94 cm in men and < 80 cm in women [1].

**Table:** 

Follow-up and surveillance	
How often should lipids be tested? - Before starting lipid treatment. - 4–12 weeks after starting therapy, or after dose modification, until the goal is achieved, then annually, unless otherwise specified.	
How often should liver enzymes (ALT) be tested? - Routinely, before treatment. - Once (8–12 weeks) after starting therapy or after dose increase. - Routine control of ALT then is not recommended during statin treatment unless previous ALT results were high or symptoms of liver disease develop. Similarly, with fibrates, control of ALT is still recommended.	
How often should muscle enzymes (CK) be tested? - Routinely, before treatment. - If CK is > 4 x ULN, do not begin the drug therapy and re-check. - Routine surveillance of CK is not recommended except if patient develops statin-associated muscle symptoms. (SAMS) (or CK increased).	
Statin-associated muscle symptoms (SAMS): Muscle pain, weakness, or aches, which occasionally occur with statin treatment, which may be associated with rise of creatine kinase (CK). Rhabdomyolysis (CK > 10 × ULN + renal injury): is the most severe form characterized by severe muscular pain, muscle necrosis, and myoglobinuria potentially leading to renal failure and death.	
In which patients should HbA1c or blood glucose be checked? Routine checks of HbA1c or glucose should be taken into consideration in patients at high risk to develop diabetes, and on high-dose statin, elderly, and patients with metabolic syndrome, obesity, or other signs of insulin resistance.	

#### Lipid-lowering drugs

##### Statins

Statins lower cholesterol levels through selective and competitive inhibition of 3-hydroxy-3-methylglutaryl-coenzyme A (HMG-CoA) reductase. They also reduce VLDL and IDL, which are LDL precursors and so they also decrease TG. High-intensity statin therapy typically lowers LDL-C levels by ≥ 50%, moderate-intensity statin therapy by 30% to 49%, and low-intensity statin therapy by < 30%. Myositis, myopathy, and elevation of the liver enzymes are the main side effects. Atorvastatin (10–80 mg), rosuvastatin (5–40 mg), simvastatin (10–40 mg), pravastatin (10–80 mg), fluvastatin (20–80 mg), and lovastatin (20–80 mg) are the main statins used [27, 28].

##### Ezetimibe

Ezetimibe inhibits cholesterol absorption at the small intestine brush lining, resulting in a decrease in intestinal cholesterol passage to the liver. The recommended dose for adults is 10 mg once daily [29].

##### PCSK9 inhibitors

PCSK9 inhibitors are fully human IgG monoclonal antibodies that selectively bind PCSK9 and prevent its binding with the LDL receptors, resulting in a reduction of LDL-C, TC, ApoB, and non-HDL-C. They decrease LDL-C levels by approximately 60%. Alirocumab and evolocumab are the currently commercially available PCSK9 inhibitors [30].

##### Bile acid sequestrants

Bile acid-binding resins bind to the main biliary acids, causing increased plasma LDL and IDL uptake. Gastrointestinal side effects are the most common. Bile acid sequestrants may delay or reduce the absorption of many drugs. So it is better taken at least 1 h before and 4–6 h after taking resins [31].

##### Omega-3 fatty acids

Omega-3 fatty acids are poly-unsaturated fatty acids that have functional and metabolic effects (reduce serum TG (through an increase in the oxidation of fatty acids)). The pharmaceutical form is represented by soft capsules of 1000 mg, containing 460–465 mg of eicosapentaenoic acid (EPA) and 375–380 mg of docosahexaenoic acid (DHA), taken twice a day. The common side effects are gastrointestinal.

##### Fibrates

Fibrates (fenofibrate, bezafibrate, ciprofibrate, and gemfibrozil) exert their effects mainly by activating the peroxisome proliferator-activated receptor alpha (PPAR-alpha) leading to a decrease in TG levels and an increase in HDL-C levels. The effect on LDL-C levels is variable. The active metabolite of fenofibrate is excreted mainly through kidneys. Combination of gemfibrozil and statins is not recommended [1, 32].

##### Niacin

Niacin raises HDL levels significantly but decreases VLDL and LDL levels. It acts mainly by preventing lipolysis in adipose tissue. The most common side effect of niacin is skin vasodilatation (flushing and itching), reversible increase in plasma levels of AST and ALT. Its combination with statins may lead to an increased risk of myopathy and rhabdomyolysis [1, 27].

##### Lomitapide and mipomersen

Lomitapide is a selective inhibitor of the microsomal triglyceride transfer protein (MTP) and reduces LDL-C levels by approximately 40%. Mipomersen is an antisense oligonucleotide that inhibits the production of apoB-100 and lowers LDL-C by approximately 37%. Both represent new therapeutic approaches for patients with homozygous FH who do not reach LDL targets with statins [33].

## Conclusion

Management algorithms proposed in this document are simple, cover the clinical situations most commonly observed in routine practice, and are designed to improve the quality of care. The strategies recommended for elevated plasma lipids are a central objective in the effort to prevent ASCVD. Encouraging healthy lifestyle is essential in each approach. Screening for cardiovascular risk is recommended for all asymptomatic adults 40 years of age or older. High-risk SCORE chart is used for risk assessment in the Egyptian population. The target is to reduce LDL-C by 50% from the baseline and reach an absolute LDL-C of < 55 mg/dL for very-high-risk patients. A high-intensity statin is prescribed up to the highest tolerated dose to reach the treatment goals; if the goals are not achieved, ezetimibe, with or without a PCSK9 inhibitor, may be considered for secondary prevention.

## Data Availability

The dataset supporting the results and conclusions of this article will be available from the corresponding author on request.

## Abbreviations

ALT: Alanine transaminase
Apo B: Apolipoprotein B
ASCVD: Atherosclerotic cardiovascular disease
AST: Aspartate transaminase
BMI: Body mass index
CAD: Coronary artery disease
CK: Creatine kinase
CKD: Chronic kidney disease
CT: Computed tomography
CV: Cardiovascular
CVD: Cardiovascular disease
DHA: Docosahexaenoic acid
DLCN: Dutch Lipid Clinic Network
DM: Diabetes mellitus
eGFR: Estimated glomerular filtration rate
EPA: Eicosapentaenoic acid
FCH: Familial combined hyperlipidemia
FH: Familial hypercholesterolemia
GFR: Glomerular filtration rate
HDL-C: High-density lipoprotein cholesterol
HeFH: Heterozygous familial hypercholesterolemia
HMG-CoA: 3-hydroxy-3-methylglutaryl-coenzyme A
HoFH: Homozygous FH
HTG: Hypertriglyceridemia
IDL: Intermediate-density lipoprotein
Ig G: Immunoglobulin G
LDL-C: Low-density lipoprotein cholesterol
LDLR: Low-density lipoprotein receptor
Lp(a): Lipoprotein (a)
MI: Myocardial infarction
MTP: Microsomal triglyceride transfer protein
PAD: Peripheral arterial disease
PCSK9: Proprotein convertase esubtilisin/kexin type 9
PCI: Percutaneous coronary intervention
PPAR alpha: Peroxisome proliferator-activated receptor alpha
SAMS: Statin-associated muscle symptoms
SCORE: Systematic Coronary Risk Estimation
SLE: Systemic lupus erythematosis
TC: Total cholesterol
TG: Triglycerides
TIA: Transient ischemic attack
T1DM: Type 1 diabetes mellitus
T2DM: Type 2 diabetes mellitus
ULN: Upper limit of normal
VLDL: Very-low-density lipoprotein
WHO: World Health Organization
